# HIF3A gene disruption causes abnormal alveoli structure and early neonatal death

**DOI:** 10.1371/journal.pone.0300751

**Published:** 2024-05-08

**Authors:** Tomoki Kawahata, Kitaru Tanaka, Kyohei Oyama, Jun Ueda, Kensaku Okamoto, Yuichi Makino

**Affiliations:** 1 Division of Endocrinology, Metabolism, and Rheumatology, Department of Internal Medicine, Asahikawa Medical University, Asahikawa, Japan; 2 Department of Cardiac Surgery, Asahikawa Medical University, Asahikawa, Japan; 3 Department of Advanced Medical Science, Asahikawa Medical University, Asahikawa, Japan; 4 Center for Integrated Medical Education and Regional Symbiosis, Asahikawa Medical University, Asahikawa, Japan; Duke University, UNITED STATES

## Abstract

Transcriptional response to changes in oxygen concentration is mainly controlled by hypoxia-inducible transcription factors (HIFs). Besides regulation of hypoxia-responsible gene expression, HIF-3α has recently been shown to be involved in lung development and in the metabolic process of fat tissue. However, the precise mechanism for such properties of HIF-3α is still largely unknown. To this end, we generated HIF3A gene-disrupted mice by means of genome editing technology to explore the pleiotropic role of HIF-3α in development and physiology. We obtained adult mice carrying homozygous HIF3A gene mutations with comparable body weight and height to wild-type mice. However, the number of litters and ratio of homozygous mutation carriers born from the mating between homozygous mutant mice was lower than expected due to sporadic deaths on postnatal day 1. HIF3A gene-disrupted mice exhibited abnormal configuration of the lung such as a reduced number of alveoli and thickened alveolar walls. Transcriptome analysis showed, as well as genes associated with lung development, an upregulation of stearoyl-Coenzyme A desaturase 1, a pivotal enzyme for fatty acid metabolism. Analysis of fatty acid composition in the lung employing gas chromatography indicated an elevation in palmitoleic acid and a reduction in oleic acid, suggesting an imbalance in distribution of fatty acid, a constituent of lung surfactant. Accordingly, administration of glucocorticoid injections during pregnancy resulted in a restoration of normal alveolar counts and a decrease in neonatal mortality. In conclusion, these observations provide novel insights into a pivotal role of HIF-3α in the preservation of critically important structure and function of alveoli beyond the regulation of hypoxia-mediated gene expression.

## Introduction

Adaptation to dynamic changes in environmental oxygen levels is essential for most species on earth. For example, the mammalian fetus experiences a transition from an intrauterine life maintained by cord blood supply to an extrauterine air-breathing life at birth entailing a drastic change of ambient oxygen tension. This involves complex physiological, anatomical, and biochemical changes in respiratory and cardiovascular systems. Emerging information has become available allowing us to understand the molecular basis underlying such adaptive responses. In particular, the transcriptional regulation of gene expression is a crucial component of the molecular response to alteration of oxygen concentration and mainly regulated by hypoxia-inducible transcription factors (HIFs).

HIF is a heterodimer consisting of oxygen-sensitive α subunits, HIF-1α, HIF-2α, and HIF-3α, and a constitutively expressed β subunit, HIF-1β. Under normoxic conditions, HIF-α is ubiquitinated by the von Hippel-Lindau (pVHL) protein and rapidly degraded by proteasome [1, 2]. On the other hand, under hypoxic conditions, pVHL is released to stabilize the HIF-α protein [3], and the stabilized HIF-α translocates to the nucleus, dimerizes with HIF-1β, and binds to the hypoxia response element of the target gene [4].

HIF-1α and HIF-2α are central to oxygen homeostasis in many physiological and pathological processes as well as developmental programs and plays overlapping but not redundant roles through the tissue-specific or temporal patterns of expression to control different transcriptional targets. In contrast, much less is known about HIF-3α. This is at least in part because of the existence of multiple HIF-3α variants. Such variants are shown to use different promoters, and different composition of the exons thus may distribute in a temporary and spatially distinct manner. Elucidation of HIF-3α function in the cellular process related to physiological and pathological events, therefore, is a great challenge.

In mice, three splicing variants forming the HIF3A gene locus have been identified: HIF-3α, neonatal and embryonic PAS protein (NEPAS), and inhibitory PAS domain protein (IPAS). HIF-3α and NEPAS are thought to be less transcriptionally active than HIF-1α and HIF-2α because they lack a C-terminal transcriptional activation domain (C-TAD). Therefore, HIF-3α and NEPAS repress the activity of HIF-1α or HIF-2α when dimerized with HIF-1β [5]. In contrast, the IPAS we identified is hypoxia-inducible [6], and its structure lacks a transcriptional activation domain [7, 8]. Previously reported HIF-3α knockout mice generated by insertion of a GFP gene into exon 2, a common exon at least in HIF-3α, NEPAS, and IPAS, showed enlargement of the right ventricle and impaired lung remodeling during development [5]. Transgenic mice that express HIF-3α, specifically in the alveolar epithelium, exhibit aberrant alveolar formation and impaired post-pseudo glandular branching in association with upregulation of several proximal lung marker genes and downregulation of the genes expressed in distal lung epithelium [9]. These results indicate the important role of the HIF3A gene in lung development.

On the other hand, in humans, the HIF3A locus is significantly hypermethylated in the adipose tissue of obese adults through genome-wide methylome analyses [10], modulates lipolysis [11], correlates with increased risk of insulin resistance and glucose metabolism [12], and indicates involvement of HIF-3α in dysregulation or maintenance of metabolic processes. A functional polymorphism in HIF-3α is related to a level of free fatty acids [13]. The overall picture of the pleiotropic HIF3A gene function however, has not been well captured.

This study aimed to determine the function of the HIF3A gene by analysis of HIF3A- gene-disrupted mice by means of genome editing technology giving less influence on the genomic structure. Surprisingly, HIF3A disruption caused acute neonatal death with alveolar structural abnormalities, and antenatal administration of glucocorticoids contributed to better survival. These results demonstrate a previously unknown function of the HIF3A gene.

## Materials and methods

### Animals

All animal experiments conformed to the National Institutes of Health Guide for the Care and Use of Laboratory Animals and were performed by a protocol approved by the Institutional Animal Care and Use Committee (IACUC)/ethics committee of the Asahikawa Medical University (Approval No. P4-032). Mice were housed in cages with free access to food and water under specific-pathogen-free conditions in rooms maintained on a 12:12 h light/dark cycle and at a temperature of 22°C and a humidity of 50%. C57BL6/J mice were purchased from Japan SLC Inc. (Shizuoka, Japan). Neonatal mice were euthanized via isoflurane anesthesia and decapitation, while adult mice were euthanized via isoflurane anesthesia and cervical dislocation. All efforts were made to minimize suffering.

### Generation of HIF3A gene-disrupted mice

For generating HIF3A gene-disrupted mice, CRISPR technology was utilized. *In vitro* fertilization (IVF) was performed as previously described [14]. Briefly, HTF (human tubal fluid) medium (ARK Resource Co., Ltd.) was used for mouse sperm preincubation, IVF, and embryo transfer. For sperm preincubation, a 200 μl droplet was used. For oocyte collection and IVF, a 100 μl volume droplet was used. Embryos were washed by passing through four such droplets. Each droplet was placed on a 35 mm culture dish (Corning® Cat. No. 430588, Thermo Fisher Scientific), covered with liquid paraffin oil (Nacalai Tesque), and kept at 37°C under 5% CO_2_ in humidified air overnight. After IVF, fertilized embryos were subjected to electroporation to inject Cas9 protein, tracrRNA and crRNA [15]. For electroporation, a platinum block electrode (Cat. No. LF501PT1-10, BEX Co., Ltd.; length: 10 mm, width: 1 mm, height: 0.5 mm, gap: 1 mm) was used. The electrode was connected to a CUY21EDIT II (BEX Co., Ltd.) electroporator, and set under a stereoscopic microscope (SZX2-ZB16, Olympus). The collected fertilized embryos cultured in HTF medium were washed with Opti-MEM™ I (Thermo Fisher Scientific) three times to remove the serum-containing medium. The embryos were then placed in a line in the electrode gap filled with 500 ng/μl Guide-it™ Recombinant Cas9 (Electroporation-Ready) protein (Takara Bio), 300 ng/μl tracrRNA and 200 ng/μl crRNA-containing Opti-MEM™ I solution (total 5 μl volume), and electroporation was performed. The electroporation conditions were 25 V (3 ms ON + 97 ms OFF) ± 3 repeats. After electroporation, the embryos were immediately collected from the electrode chamber and subjected to three washes with M2 medium (ARK Resource) followed by two washes with KSOM medium (ARK Resource). The embryos were then cultured in KSOM medium at 37°C and 5% CO_2_ in humidified air overnight, and 2-cell embryos were transferred to pseudopregnant recipient ICR female mice (Japan SLC).

The sequence targeting HIF3A gene was designed using CRISPRdirect (https://crispr.dbcls.jp/) [16] and was as follows: HIF3A (5’- GAA GGA GAA GUC GCG GGA CG-3’). The tracrRNA and crRNA were purchased from FASMAC Co. Ltd. in dry form, dissolved in Opti-MEM™ I to 1 μg/μl and stored at –30°C until use. To identify founders, tail DNA sequences were determined by a BigDye^TM^ Direct Cycle Sequencing kit (Applied Biosystems^TM^, Waltham, MA, USA) using the primers shown in S1 Table. HIF3A gene-disruption mice used in this experiment were produced by backcrossing five or more generations.

### Genotyping

Mouse genomic DNA was extracted from the tail tip using DNA extraction buffer (50 mM KCl, 1.5 mM MgCl2, 0.1% Gelatin, 0.45% Tween-20, 0.45% NP-40, 0.2 mg mL proteinase K, 10 mM Tris-HCl, pH 8.3). PCR was performed in cycles of initial denaturation at 95°C for 2 min, denaturation at 95°C for 15 sec, annealing at 60°C for 10 sec, and extension at 72°C for 20 sec. PCR primers are listed in S1 Table.

### Sequencing of HIF3A transcript variants

Total RNA was extracted from the fetal livers of each group using the RNeasy plus mini kit (Qiagen, Tokyo, Japan) according to the manufacturer’s protocol. cDNA synthesis was performed using one μg of total RNA with SuperScript™ IV VILO (Invitrogen, Waltham, MA, USA). PCR was performed with primers specific to each HIF-3α isoform using each cDNA sample as a template. Primer sequences used in this study are listed in S1 Table. The PCR products were purified with NucleoSpin Gel and PCR clean-up (TaKaRa, Shiga, Japan). Those cleaned-up PCR products were sequenced with a BigDye^TM^ Terminator v3.1 Cycle Sequencing Kit (Applied Biosystems^TM^, Waltham, MA, USA).

### Morphological study of the lungs

Isolated lung tissue was fixed in 4% paraformaldehyde/phosphate buffered saline and paraffin-embedded. Four μm sections were prepared for hematoxylin-eosin staining, and immunohistochemical staining.

Distance interalveolar space was measured using ImageJ/Fiji for all septa within five randomly selected regions. The mean alveolar septum thickness was compared among five independent neonates of each genotype.

For immunohistochemistry, deparaffinized sections were incubated overnight at 4°C with rabbit polyclonal anti Ki67 antibody (1:400) (NB500-170; Novus Biologicals, Centennial, CO, USA), rabbit polyclonal anti-PDPN antibody (1:1000) (ab11936; Abcam, Cambridge, UK), rabbit polyclonal anti-Uteroglobin/CC10 antibody (1:2000) (10490-1-AP; Protintech, Rosemont, IL, USA), rabbit polyclonal anti pro SP-C antibody (1:1000)(AB3786; Millipore, Darmstadt, Germany) and rabbit monoclonal anti-SCD1 antibody (1:100) (#2794; Cell Signaling Technology, Danvers, MA, USA). All reactions were performed using the VECTASTAIN Elite ABC kit (Vector Laboratories, Newark, CA, USA) and ImmPACT DAB Substrate kit (Vector Laboratories, Newark, CA, USA) according to the manufacturer’s protocol. Airspaces were counted in the area surrounded by anti-PDPN antibody staining. The number of airspaces per 1000 x 1000 mm^2^ of randomly selected surface area in each section was counted and compared in at least three independent neonates of each genotype. For semiquantitative analysis, SCD1-positive or Ki67-positive cells and total cells were counted per 100 x 100 mm^2^ of a randomly selected area in each section; ratios were calculated by dividing the number of SCD1-positive or Ki67-positive cells by the total cell count.

### RNA-seq

Total RNA was extracted from the lungs of postnatal day 0 (P0) of each group using the RNeasy plus mini kit (Qiagen, Tokyo, Japan) according to the manufacturer’s protocol; RNA quality was checked with a 4200 TapeStation (Agilent, Santa Clare, CA, USA). Library construction and sequencing were performed using a commercial service (Veritas Genetics, Danvers, MA, USA). FastQC (ver. 0.11.8) was used to perform quality checks on all reads, and low-quality read removal was performed using Trim Galore (ver0.6.4). STAR (ver. 2.7.0f) was used to map the mouse genome (mm10) assembly. Mapping was performed using the default values of STAR. In addition, counts were calculated for each gene defined by gene symbols using featureCount (ver. 1.6.4). Normalization was performed to align the total number of reads to one million for all samples analyzed (CPM, counts per million). Differences in gene expression were calculated using edgeR (ver. 3.22.3). The genes with Fold Change greater than 2 and a p-value less than 0.01 were defined as up-regulated. Similarly, the genes were defined as down-regulated if the Fold Change was smaller than 0.5 and the p-value was smaller than 0.01. Based on the comparative analysis of wild-type (WT) and HIF3A_ins and WT and HIF3A_del at P0, a gene list of upward and downward-regulated genes common to both was created. Additionally, separate lists of upward and downward-regulated genes were created for the comparisons of WT with HIF3A_ins and WT with HIF3A_del, respectively.

### Quantitative RT-PCR

Total RNA was extracted from the lungs of P0 of each group using the RNeasy plus mini kit (Qiagen, Tokyo, Japan) according to the manufacturer’s protocol. Five hundred ng of total RNA was converted to cDNA using SuperScript^TM^ IV VILO Master Mix (Invitrogen, Waltham, MA, USA). Quantitative PCR was performed using 2 x TaqMan Master Mix and TaqMan primer probes (Applied Biosystems^TM^, Waltham, MA, USA): stearoyl-Coenzyme A desaturase 1 (SCD1) gene (assay ID: Mm00772290), claudin 6 (Cldn6) gene (assay ID: Mm01309194), chymotrypsin-like elastase family, member 1 (Cela1) gene (assay ID: Mm00712898), hypoxia inducible factor 1a (assay ID: Mm00468869), endothelial PAS protein 1 (assay ID: Mm01236112), actin, beta (Actb) gene (assay ID: Mm01205647). For quantitative analysis, the amount of cDNA in each sample was normalized against the level of the housekeeping gene Actb using the comparative CT method.

### Fatty acid composition

Lipids were extracted from the lungs of P0 of each group by the Bligh and Dyer method [17]. Briefly, lung tissue was homogenized in a methanol/chloroform (2:1, v/v) solution; after centrifugation at 2,000 rpm for 5 minutes, the supernatant was collected. Distilled water/chloroform (1:1, v/v) solution was added to the collected supernatant; after centrifugation at 2,000 rpm for 5 min, the upper layer was discarded, and the chloroform layer was transferred to a test tube. The chloroform layer was concentrated in a centrifugal evaporator, and the obtained lipids were analyzed by gas chromatography at a commercial service (SRL Inc., Fukuoka, Japan).

### Antenatal glucocorticoid treatment

Heterozygous females and males were mated at a fixed time. Female mice were checked for mating plugs and designated as gestation day 0.5. All females were individually separated and housed after the mating plug was confirmed. Pregnant mice were randomly divided into two groups. In the treatment group, 0.1 mg betamethasone (SHIONOGI & CO., LTD., Osaka, Japan) dissolved in sterile saline was subcutaneously administered to the mice on days 17.5 and 18.5 of gestation. The control group received the same volume of sterile saline without betamethasone on the same days.

### Statistical analysis

All data are presented as means ± SD or SEM from the indicated repeated experiments. Survival rate at P1 was analyzed using Fisher’s exact test or the Mann-Whitney U test. For determining significant differences in multiple comparisons, statistical analysis was performed by one-way ANOVA followed by Tukey’s or Dunnet post hoc analysis or by the Kruskal-Wallis test followed by a Dunn test. A P-value < 0.05 was considered statistically significant. All statistical analyses were performed using GraphPad Prism (ver. 7.0; San Diego, CA, USA).

## Results

### Generation of HIF3A gene-disrupted mice

**Fig 1:**
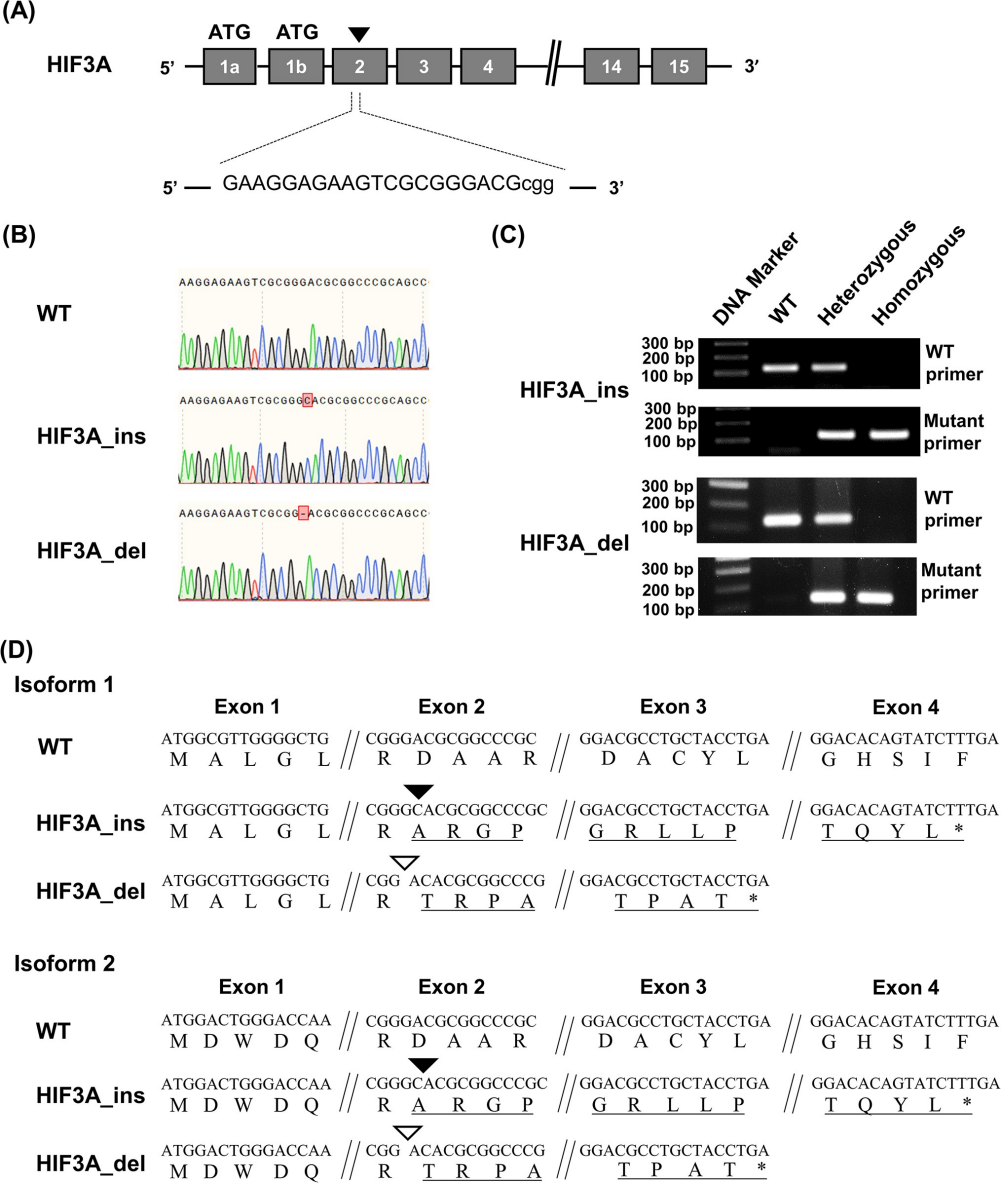
Generation of HIF3A gene-disrupted mice through genome editing. (A) A representative view of the CRISPR/Cas9 targeting strategy used for HIF3A gene-disrupted mice. Sequences of gRNA targeting exon2, a common exon for all transcripts, are shown. The triangle indicates the target site. PAM sequences are lowercase letters. (B) Confirmation of genomic DNA mutations. DNA extracted from tails of wild-type (WT) and mutant-allele mice were analyzed by Sanger sequencing. There were two types of mutations: a C insertion (HIF3A_ins) and a G deletion (HIF3A_del) in exon2. (C) Detection of mutant alleles. PCR was performed on genomic DNA extracted from mice tails using primers to detect the WT allele and the respective mutant allele. The molecular weight marker shows a 100 bp ladder. (D) Confirmation of premature termination codons by a frameshift. Total RNA was extracted from fetal livers homozygous for the mutant allele, and RT-PCR was performed. The top row shows the nucleotide sequence, and the bottom row shows the amino acid sequence. The black triangle indicates the site where C has been inserted, while the white triangle shows the site where G has been deleted.

To generate HIF3A gene-disrupted mice, we employed CRISPR/Cas9-directed genome editing technologies in the fertilized eggs of C57BL/6J mice. The guide RNA to introduce DNA break included a 20 nucleotides sequence targeting exon2, a common exon among splicing products of the HIF3A gene, and a protospacer adjacent motif (Fig 1A). The gRNA and Cas9 were co-injected into fertilized eggs, and live-born mice were obtained. Sanger sequencing of genomic DNA extracted from the tails of these mice confirmed that the mice had a single nucleotide insertion (HIF3A_ins) or deletion (HIF3A_del) in exon 2 (Fig 1B). Actually, PCR using primers annealing to each of these mutated sequences distinguish the wild-type (WT) allele from the corresponding mutant allele (Fig 1C). These mutations were predicted to yield premature termination codons (PTCs) by frameshifts in the gene transcripts and thus disrupt gene production formation. To confirm the PTCs, we first generated cDNA from the total RNA of genome-edited mice using random primers for reverse transcription, then PCR was performed to amplify cDNA corresponding to HIF3A isoform 1 (NM_001162950.1) and isoform 2 (NM_016868.3) by employing isoform-specific forward primer annealing to exon1a and exon1b, respectively, and a common reverse primer annealing to exon15. Sequencing of the PCR products revealed frameshifts in both isoforms that caused PTCs (Fig 1D). Except for the indel sites, the coding sequences of both isoforms of WT, HIF3A_ins and HIF3A_del mutant mice were identical (S1 Appendix). These data indicate that we have obtained two different types of HIF3A gene-disrupted mice, HIF3A_ins and HIF3A_del. In addition, we demonstrated Custom TaqMan™ SNP Genotyping Assay to show in HIF3A_ins or HIF3A_del mice the WT allele-specific transcript was not detectable (S5 and S6 Figs).

### Half of HIF3A gene-disrupted mice die within one day after birth

**Fig 2:**
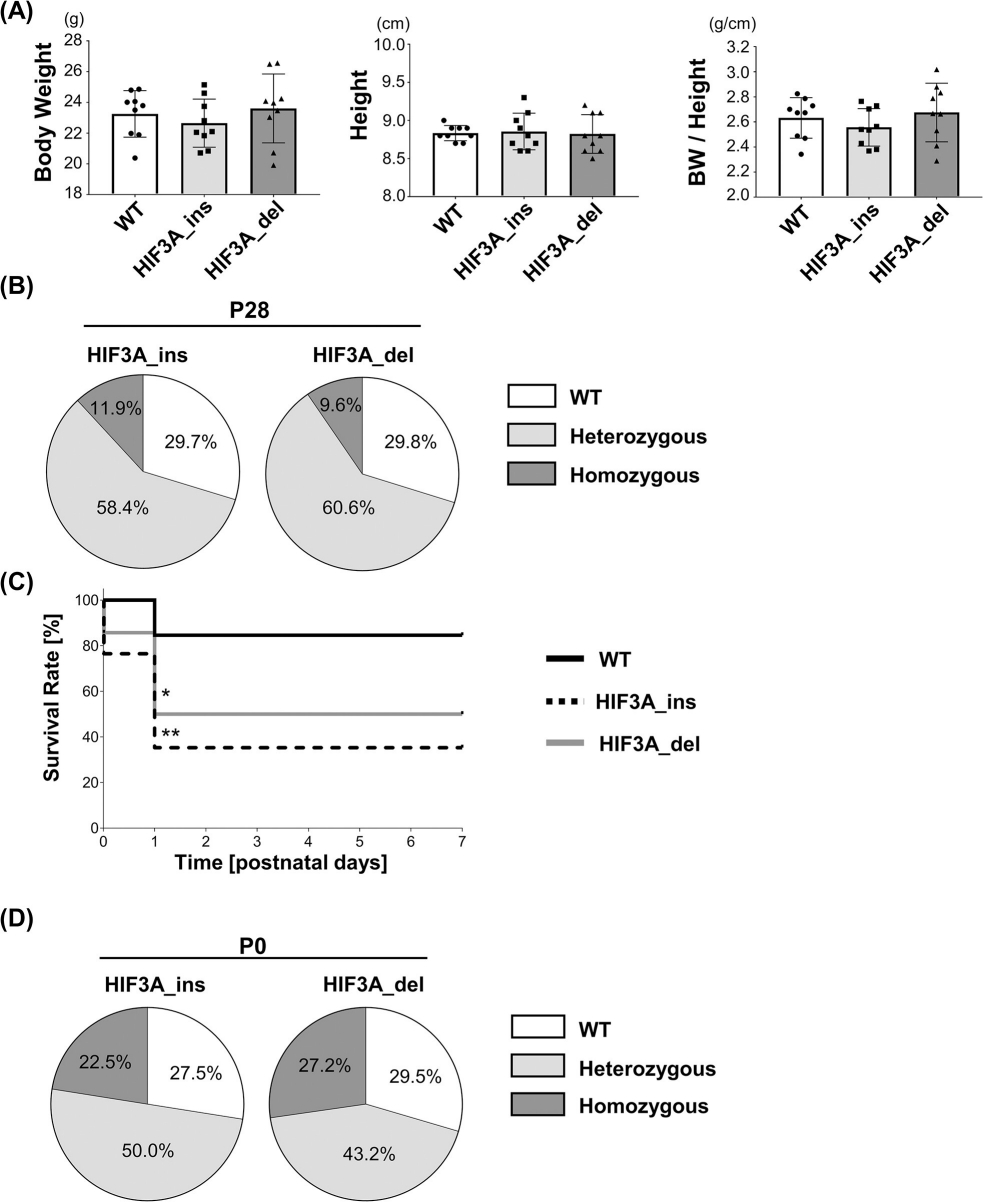
HIF3A gene-disrupted mice are viable, but some die within one day of birth. (A) Body size of 12-week-old male HIF3A gene-disrupted mice. Height was measured from nose to anus. Each data point represents values from an individual mouse. The results are presented as the mean ± SD. (B) Genotype distribution of the mice on P28. Neonatal mice were obtained by mating heterozygous male and female HIF3A_ins and HIF3A_del, respectively. n = 101 (HIF3A_ins) and 94 (HIF3A_del) mice at P28. (C) Survival curves until 7 days postnatally. Twenty-six WT and 17 HIF3A_ins and 14 HIF3A_del mice were obtained. Survival rates at postnatal day 1 were 84.6% in WT, 35.3% in HIF3A_ins and 50% in HIF3A_del (WT vs HIF3A_ins: p = 0.0025, WT vs HIF3A_del: p = 0.029, HIF3A_ins vs HIF3A_del: p = 0.48). *p < 0.05, HIF3A_del vs WT. **p < 0.01, HIF3A_ins vs WT. (D) Genotype distribution of the mice on P0. Neonatal mice were obtained by mating heterozygous male and female HIF3A_ins and HIF3A_del, respectively. n = 40 (HIF3A_ins) and 44 (HIF3A_del) mice at P0, respectively.

The mice carrying homozygous HIF3A gene mutation HIF3A_ins and HIF3A_del were viable to adulthood and fertile, with no significant differences in body weight or height compared to WT mice (Fig 2A). However, the average number of litters born from the mating between homozygous mutant mice was 3.9 ± 1.7 (HIF3A_ins) and 4.4 ± 1.5 (HIF3A_del), which were lower than the previously reported average litter sizes of 5.5 to 6.0 for C57BL/6J [18, 19]. Unexpectedly, on postnatal day 28, the genotypes of mice obtained by mating between heterozygous mutant mice deviated from the Mendelian ratio showed fewer homozygous mutations; 11.9% for HIF3A_ins mice and 9.6% for HIF3A_del mice, although both homozygotes were expected to be 25% (Fig 2B). Exploration of the survival rate of newborn mice over 7 days showed that survival at postnatal day 1 was significantly reduced in HIF3A_ins to 35.3% and HIF3A_del to 50% compared to 84.6% for the WT (WT vs HIF3A_ins: p = 0.0025, WT vs HIF3A_del: p = 0.029), while there was no significant difference between survival rates for HIF3A_ins and that for HIF3A del (p = 0.48) (Fig 2C). No further deaths occurred afterwards. Importantly, the genotypes analysis at postnatal day 0 (P0) of the offspring from mating between heterozygous mutant mice exhibited the composition of the genotypes that followed the Mendelian ratios (Fig 2D). Taken together, some portion of the HIF3A gene-disrupted mice had a phenotype of sudden death within the first day after birth.

### HIF3A gene-disrupted mice exhibit abnormal lung structures

**Fig 3:**
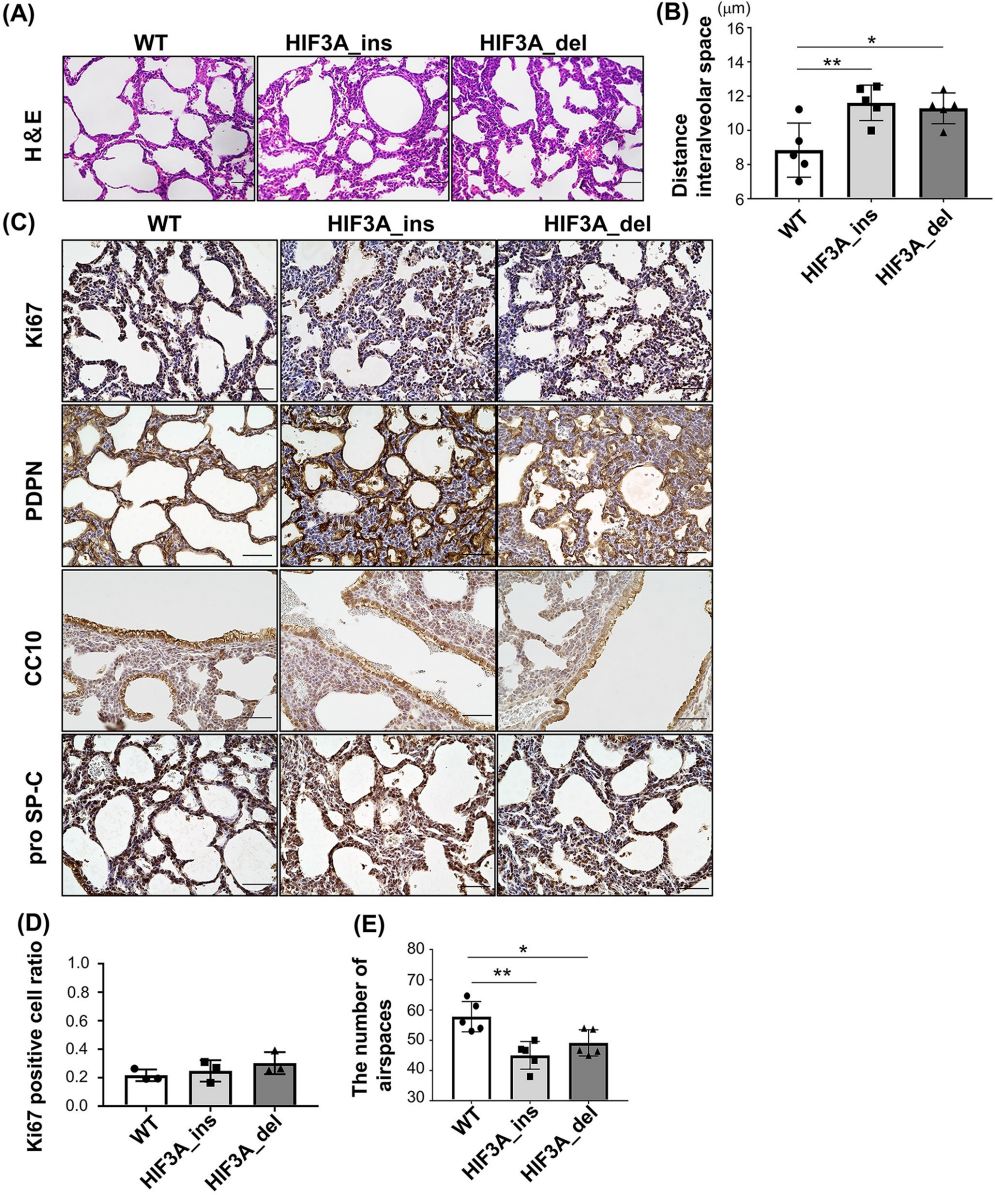
Lung structures of HIF3A gene-disrupted mice at P0. (A) Hematoxylin and eosin staining of lungs at P0 are shown. Scale bar indicates 50 μm. (B) The distance between interalveolar spaces was measured semi-quantitatively. The results are presented as the mean ± SD. Comparisons were analyzed by one-way ANOVA, Tukey’s post hoc test (n = 5). (C) Immunohistochemistry for Ki67, podoplanin (PDPN), CC10 and prosurfactant Protein C (pro SP-C) staining. Scale bars indicate 50 μm. (D) Semiquantitative analysis of Ki67-positive cell counts. Ratios were calculated by dividing Ki67-positive cells from the total cell count. The results are presented as the mean ± SD. Comparisons were analyzed by Kruskal-Wallis test followed by Dunn test. (n = 3). (E) Semiquantitative analysis of the number of spaces surrounded by PDPN (number of alveoli). The results are presented as the mean ± SD. Comparisons were analyzed by one-way ANOVA, Tukey’s post hoc test (n = 5). *p < 0.05, **p < 0.01.

As a cause of death within one day of birth, we speculated unsuccessful oxygen intake via pulmonary respiration after termination of umbilical blood supply, and thus assessed lung structure at birth. At P0, thinning of alveolar septa and mesenchyme, which is essential for neonatal blood gas exchange, scarcely occurred in newborns of HIF3A gene-disrupted mice (Fig 3A and 3B). No differences were observed in the proliferation of epithelial or mesenchymal cells between WT and HIF3A gene-disrupted lungs as indicated by Ki67 staining (Fig 3C and 3D). The number of alveoli was determined by a numeration of the spaces surrounded by podoplanin (PDPN), a marker of type I alveolar epithelium, which revealed a significantly reduced number of alveoli in both lines of HIF3A gene-disrupted mice compared to WT (Fig 3C and 3E). These results suggest that disruption of the HIF3A gene causes abnormal alveolar configuration immediately after birth. On the other hand, there was no difference in immunohistochemical distribution of CC10 and prosurfactant Protein C (pro SP-C) between WT, HIF3A_ins, HIF3A_del mice, indicating differentiation of Clara cells and type II alveolar epithelium were not affected by HIF3A gene mutations (Fig 3C).

We also investigated vessels in the alveoli as explored by Yamashita et al. [5]. In particular, elastic fibers surrounding vessels in the lung were stained by means of Elastica-van Gieson staining and vessels were evaluated regarding the number of elastic membrane layers. Vessels with diameters less than 30 μm were divided into three categories: vessels with a single elastin fiber (SEF), vessels with partially multiple elastin fibers (PEF), and vessels with fully multiple elastin fibers (MEF). MEFs were defined as muscular arteries based on the presence of internal and complete external elastic lamina [20]. HIF3A-gene disrupted mice tended to have more MEF compared to WT (S7 Fig), suggesting muscular arterialization of small vessels in lungs in mice with HIF3A gene disruption.

### Impact of HIF3A gene disruption on global gene expression in the neonatal lung

**Fig 4:**
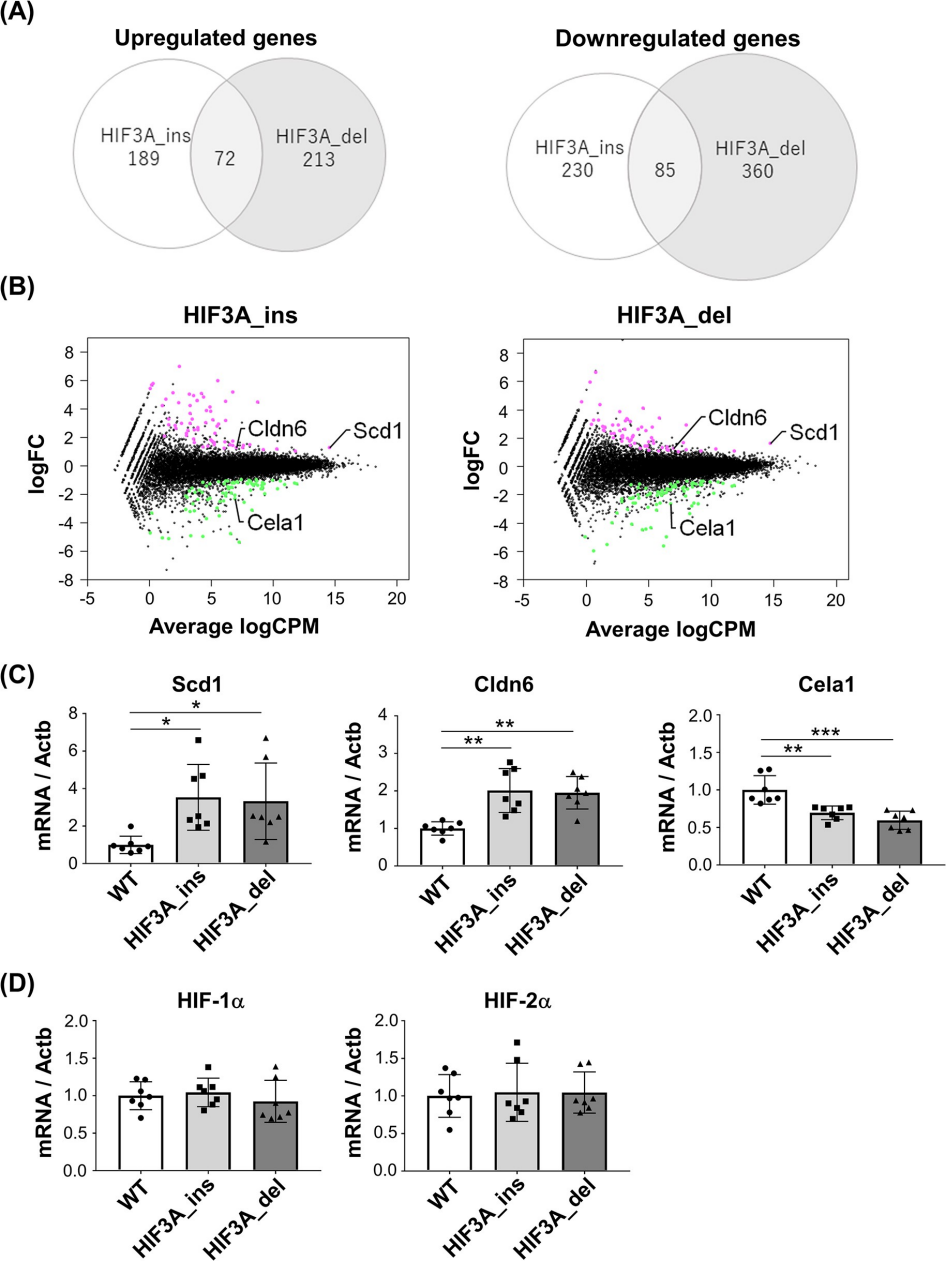
Global gene expression profiles in HIF3A gene-disrupted neonatal mice in lungs. (A) Venn diagram of the number of genes whose expression was significantly up-regulated (left panel) or downregulated (right panel) in HIF3A_ins and HIF3A_del mice at P0, respectively. Total RNA was isolated from lungs and RNA-seq was performed (n = 6 each). Genes with more than 2-fold or less than 0.5-fold with P<0.01 compared to HIF3A_ins and HIF3A_del, with WT mice as control, were defined as significantly altered expression genes. (B) MA plot. HIF3A_ins and HIF3A_del were compared with WT mice, respectively. Each dot represents the average expression level (log2 CPM) on the X-axis and fold change (FC) (log2 FC) on the Y-axis of the gene encoding the protein. Magenta dots represent genes that are differentially up-regulated in HIF3A gene deletion mice compared to WT mice, and green dots represent genes that are differentially up-regulated. mRNA expression levels of (C) representative differentially expressed genes, (D) HIF-1α and HIF-2α from quantitative real-time PCR analysis. The results are presented as the mean ± SD. Comparisons were analyzed by one-way ANOVA, Tukey’s post hoc test (n = 7 each). *p < 0.05, **p < 0.01, ***p < 0.001.

We hypothesized that lung abnormalities in HIF3A gene-disrupted mice might contribute to death immediately after alveolar ventilation. To assess the impact of HIF3A gene disruption on global gene expression in the lung, RNA-seq was performed on the lung specimens of HIF3A_ins, HIF3A_del, and WT mice at P0 (n = 6 each). In the differential gene expression analysis, we compared HIF3A_ins and HIF3A_del against WT and defined a gene as significant if it changed more than 2-fold or less than 0.5-fold with a P value <0.01. There were 72 genes that were commonly up-regulated and 85 genes that were commonly down-regulated in HIF3A_ins and HIF3A_del compared to WT (Fig 4A). The list of genes with significantly different expression in S2 and S3 Tables, and results of the gene ontology analyses are in S1 and S2 Figs. The results of pathway analyses are shown in S3 and S4 Figs.

Among those commonly regulated genes in both mutant mice, we found three genes in the chymotrypsin-like elastase family: member 1 (Cela1), claudin 6 (Cldn6), and stearoyl-Coenzyme A desaturase 1 (SCD1) (Fig 4B). Cela1 is known to cause lung remodeling dysfunction [21–23] and Cldn6 contributes to abnormal lung development [24, 25]. SCD1 is known to play a role in fatty acid metabolism. Of note, SCD1 showed a high level of expression and high magnitude of change in the expression level, and thus seemed to have an apparent influence on the phenotype of the lung of those mutant mice (Fig 4B). Moreover, a previous report showed down regulation of SCD1 in neonatal mice with alveolar epithelium-specific overexpression of HIF-3α [9], indicating that manipulation of the HIF3A gene may closely associate with altered SCD1 gene expression. Quantitative PCR for those three genes supported the results of RNA-seq (Fig 4C). In contrast, HIF-1α and HIF-2α mRNA levels were not significantly different (Fig 4D). Collectively, HIF3A gene disruption affects a wide variety of gene expression in the lung.

### Disruption of HIF3A gene alters fatty acid composition in lung

**Fig 5:**
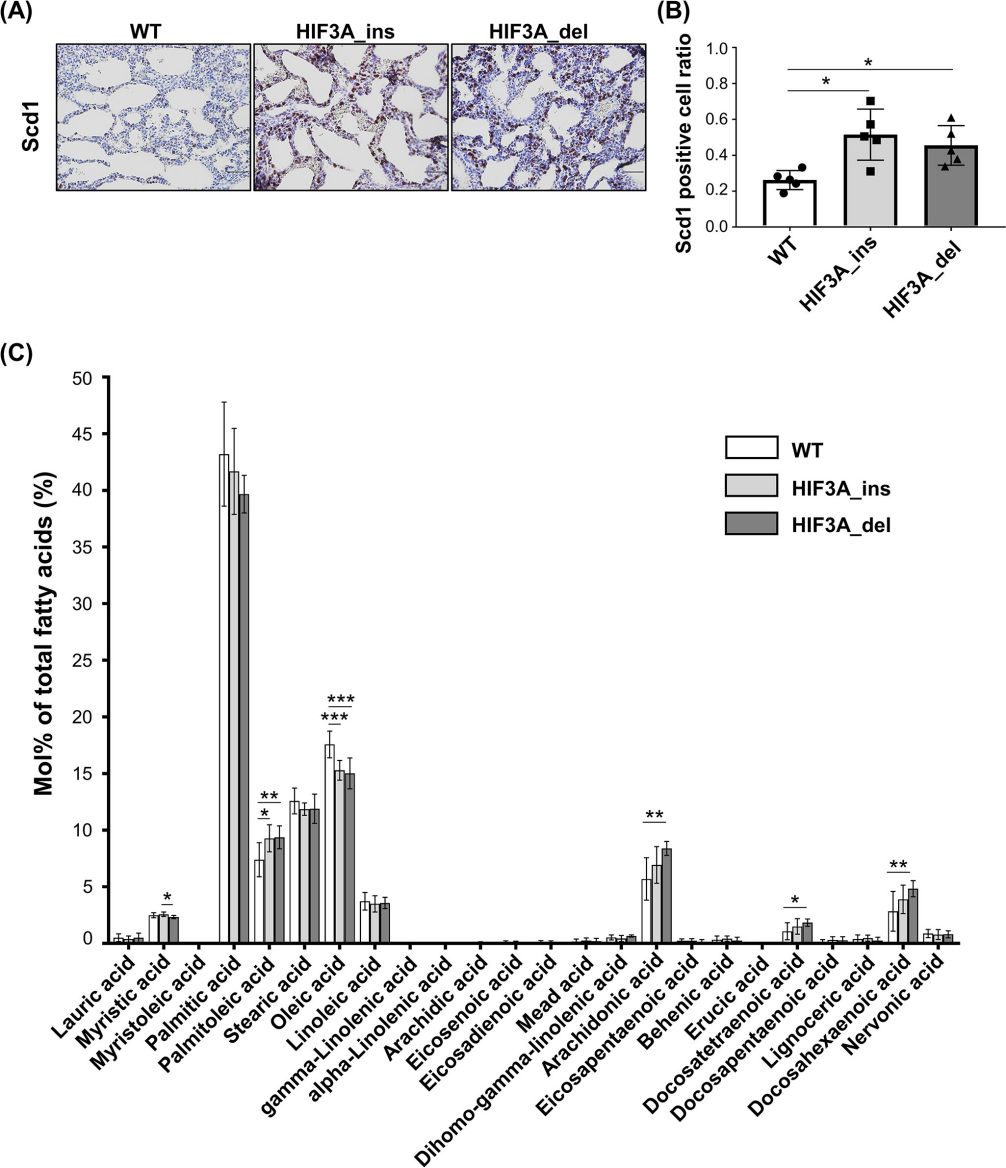
Fatty acid metabolism in lungs of HIF3A gene-disrupted mice. (A) Representative images of immunohistochemical staining of SCD1 in neonatal lungs. Scale bar indicates 50 μm. (B) Semiquantitative analysis of SCD1-positive cell counts. Ratios were calculated by dividing SCD1-positive cells from the total cell count. The results are presented as the mean ± SD. Comparisons were analyzed by Kruskal-Wallis test followed by Dunn test. (n = 5). *p < 0.05. (C) Fatty acid composition in lungs of neonatal mice at P0. Total lipids were extracted from lungs and analyzed by gas chromatography. The results are presented as the mean ± SD. All comparisons were analyzed by one-way ANOVA, Tukey’s post hoc test (n = 9 each). *p < 0.05, **p < 0.01, ***p < 0.001.

Given the RNA-seq results, we performed immunohistochemical analyses to confirm the localization and level of expression of SCD1 in the lungs in HIF3A gene-disrupted mice. SCD1 was expressed mainly in the alveolar epithelium and those SCD1-positive cells were found at a significantly higher frequency in HIF3A_ins and HIF3A_del mice than in WT mice. (Fig 5A and 5B). SCD1 is a rate-limiting enzyme that converts saturated fatty acids to monounsaturated fatty acids; e.g., palmitic acid and stearic acid to palmitoleic acid and oleic acid, respectively [26]. In this line, we examined fatty acid distribution in the lung of HIF3A gene-disrupted mice at P0. Total lipids were extracted from the lungs, and fatty acid composition was determined by gas chromatography. Interestingly, the relative amount of palmitoleic acid was significantly elevated and that of oleic acid was significantly lower in HIF3A_ins and HIF3A_del compared to WT mice (Fig 5C). These results indicate that disruption of the HIF3α gene affects fatty acid distribution in the lungs.

### Betamethasone therapy prevents neonatal death of HIF3A gene-disrupted mice

**Fig 6:**
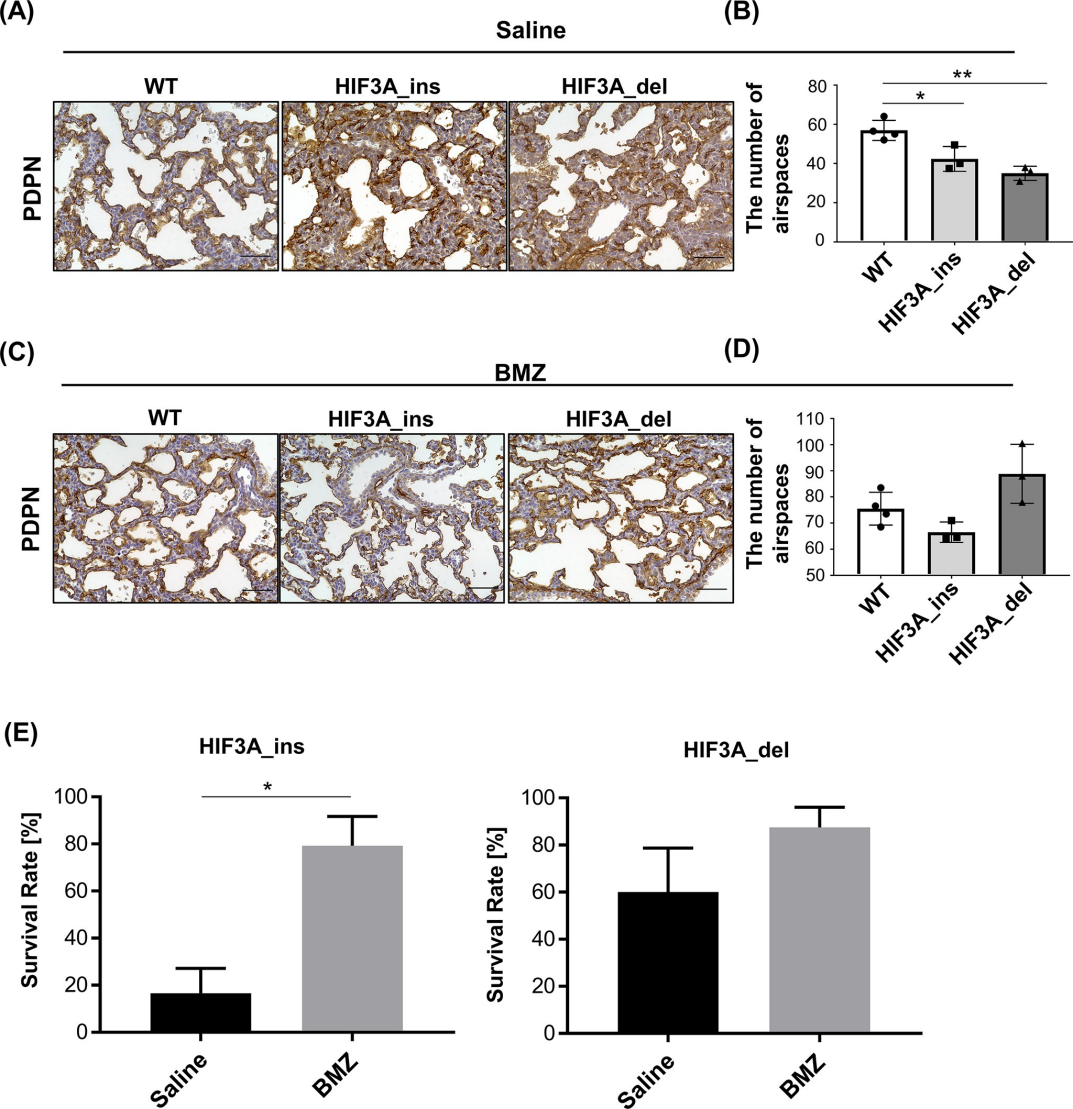
Glucocorticoid treatment prevents neonatal death of HIF3A gene-disrupted mice. (A, C) Representative images of immunohistochemical staining for podoplanin (PDPN) in the lungs of neonatal mice born from pregnant mice after saline (A) and betamethasone (C) administration are shown. Scale bar indicates 50 μm. (B, D) The number of spaces surrounded by PDPN (number of alveoli) was analyzed semiquantitatively. The results are presented as the mean ± SD. Comparisons were analyzed by one-way ANOVA, Dunnet post hoc test (n ≥ 3). *p < 0.05, **p < 0.01. (E) Survival rate of HIF3A_ins (A) or HIF3A_del (B) after betamethasone or saline treatment. The mean survival rates of WT and homozygous newborns for each pregnant mouse were compared. The results are presented as the mean ± SEM. Comparisons were analyzed by the Mann-Whitney U test (n ≥ 4). *p < 0.05.

Fatty acids are a component of the lung surfactant [27], and impaired lung surfactant function is known to cause respiratory distress leading to neonatal death. In a clinical setting, glucocorticoids are some of the most commonly used drugs for patients with respiratory distress syndrome in adults and infants and are associated with a reduction in patient mortality. Antenatal glucocorticoid treatment can prevent surfactant deficiency causing infant respiratory distress [28].Therefore, we determined the effect of administration of synthetic glucocorticoids such as betamethasone on the alveolar structure and survival rate in the mutant mice. To this end, we injected betamethasone and saline as a control to E17.5 and E18.5 pregnant mice carrying the heterozygous gene mutation of HIF3A_ins and HIF3A_del, and morphological evaluation of the lungs at P0 was performed. Both HIF3A_ins and HIF3A_del mice in the saline group demonstrated a decrease in the number of alveoli compared to WT mice. In contrast, in the betamethasone group, the number of alveoli in both mutant mice seemed comparable to that of WT (Fig 6A–6D). In addition, we examined the survival in mutant mice after betamethasone treatment. Betamethasone treatment significantly increased the survival rate of HIF3A_ins (16.6±10.5% in the saline group and 79.3±16.6% in the betamethasone group: p = 0.024). Similarly, the survival rate of HIF3A_del was increased from 60.0 ± 18.7% to 87.5 ± 8.5% by betamethasone treatment, although the difference was not statistically significant (p = 0.31). This suggests that a lethal condition in neonatal HIF3A gene-disrupted mice might be partially ameliorated by betamethasone administration. Taken together, HIF3A gene disruption might cause neonatal death due to alveolar disorders that can be prevented by glucocorticoid administration.

## Discussion

Lungs of newborn mice with HIF3A gene disruption exhibit thickened alveolar septal walls and reduced alveoli. These histological changes may suggest a presence of impaired alveogenesis. The alveolar septum consists of alveolar epithelial cells, vascular endothelial cells, and fibroblasts [29]. The thickening of the alveolar septal wall in HIF3A gene-disrupted mice was not due to hyperplasia, as Ki67 staining, an indicator of active phases of the cell cycle [30], was similar in HIF3A gene-disrupted and WT mice. Through transcriptome analysis, we found that the Cldn6 gene was significantly increased, and the Cela1 gene was decreased in HIF3A gene-disrupted mice. Sustained elevation in expression of Cldn6 in the alveolar epithelium is reported to associate with delayed lung development including reduced air space and thickened alveolar septum [24]. Regarding Cela1, gene silencing by shRNA in the MFLM4 cell line, derived from mouse fetal lung mesenchyme, inhibits angiogenesis, a process necessary for alveolus formation [22]. Therefore, Cldn6 and Cela1 might play a part in the immaturity of the alveolar structure, such as thickening of alveolar septa. Immature alveolar structure might prevent creation of proper lung compliance and efficient gas exchange insufficient to maintain the life of newborns immediately after cessation of oxygen supply via cord blood [31]. On the other hand, the reduced number of alveoli observed in neonates with HIF3A gene disruption may represent alveoli collapse. Alveolar collapse is a cause of respiratory distress syndrome and known to link to abnormal function of pulmonary surfactants. Of note, neonatal HIF-2α knockout mice develop respiratory distress syndrome and die within hours due to impaired surfactant production by type 2 alveolar epithelium [32]. Alveolar epithelial-specific HIF-1α deficient mice suffer from respiratory distress syndrome with thickened alveolar septum and die within hours [33]. These facts indicate the lack of HIFα negatively impacts the preservation of alveolar structure and function. In the present study, however, expression of HIF-1α and HIF-2α were unaffected in HIF3A gene-disrupted mice compared to the WT mice, suggesting that the lung disorder of HIF3A gene-disrupted mice is not caused by a mechanism mediated by HIF-1α or HIF-2α. Interestingly, previous HIF3A gene knockout mice employing insertion of GFP gene into exon2 of HIF3A, showed incomplete alveolar spaces in P15 and adults [5]. Moreover, the mice modified to express HIF-3α solely in the alveolar epithelium exhibited an abnormal alveolar structure, and retinoic acid receptor beta, a protein responsible for alveoli formation, was increased [9]. In addition, in the present study, we demonstrated muscularization of vessels in alveoli of HIF3A gene-disrupted mice as shown in HIF3A gene knockout mice by Yamashita et al. [5]. These results indicate that maintaining adequate HIF-3α expression is crucial for preserving normal alveolar structure and normal vascular structure in alveoli. We did not see any structural abnormality in the heart such as enlargement of the right ventricle (S8 Fig), and elevation of endothelin-1 expression as Yamashita et al. previously reported [5]. Although it is difficult at this moment to hypothesize about phenotypical differences in these HIF3A gene knockout mice, a distinct way of gene disruption, e.g. GFP-insertion vs insertion/deletion of a single nucleotide, might cause those differences.

We performed maternal glucocorticoid administration to examine the effect of glucocorticoid on neonatal deaths of HIF3A gene-disrupted mice. It has been documented that prenatal glucocorticoid therapy increases fetal pulmonary surfactant production and thins alveolar septal walls, thereby preventing respiratory failure and reducing the risk of life-threatening conditions at birth [28, 34, 35]. These effects are particularly important for preterm infants with immature lung development. We found glucocorticoid administration resulted in the preservation of a greater number of alveoli in HIF3A gene-disrupted mice compared to WT. This observation suggests that glucocorticoids prevented a loss of alveolar space, which may relate to an amendment of pulmonary surfactant dysfunction. The pulmonary surfactant consists of pulmonary surfactant protein, phospholipids, triglycerides, cholesterol, and fatty acids [27]. Of interest, we observed alteration in the composition of fatty acids, an important substrate for surfactant phospholipid production, such as palmitoleic acid and oleic acid in the lung of HIF3A gene-disrupted mice, indicating that loss of the HIF3A gene may lead to qualitative changes in the lung surfactants. Instead of measuring the fatty acid composition of the entire lung tissue as in the present study, precisely focused measurement of the composition of the alveolar surface may provide more insight into the mechanism underlying alveolar collapse by HIF3A gene disruption.

Recent studies have shed light on the association between HIF-3α and lipid metabolism in adipocytes. As mentioned above, a genome-wide analysis shows a connection between the methylation of the HIF3A locus in adipose tissue and adult obesity [10, 36]. Silencing HIF-3α in the mouse progenitor adipocyte cell line 3T3-L1 leads to de novo synthesis of fatty acids [37]. We demonstrated in the present study an alteration in the distribution of fatty acids in the lung tissue. The observation indicated that the HIF3A gene had the potential to affect lipid metabolism in pulmonary adipocytes. A cue to elucidate the mechanism for HIF-3α-mediated lipid metabolism is the increase in SCD1 expression by HIF3A gene disruption. In accordance, SCD1 expression is reduced in the lung tissue of neonatal mice overexpressing HIF-3α in alveolar epithelium [9], implying that HIF-3α influences SCD1 gene regulation. Interestingly, it is reported HIF2α upregulates SCD1 via downregulation of PPARα under hypoxic condition [38]. We speculate removal of the dominant-negative effect of HIF3α on hypoxia-inducible gene expression by HIF3A gene-disruption could be, at least in part, a crucial molecular mechanism in upregulation of SCD1 expression. It warrants further investigation regarding the unknown function of HIF-3α, e.g. the control of lipid metabolism, utilizing HIF-3α knockout cell, in upcoming studies.

HIF3A gene disruptions using genome editing techniques that had a minimal impact on genome structure caused abnormal lung fatty acid distribution and alveolar damage resulting in acute mortality in mice, that could be prevented with antenatal administration of glucocorticoids. This study offers new insights into the role of HIF-3α in preserving the structure and function of the alveoli.

## Supporting information

S1 Table Primer list. (XLSX)

S2 Table Significantly upregulated-gene list in RNA-seq. The first sheet lists differentially upregulated genes in both HIF3_ins and HIF3A_del compared to WT. The second and third sheets, respectively, list differentially upregulated genes in HIF3A_ins and HIF3A_del compared to WT.
(XLSX)

S3 Table Significantly downregulated-gene list in RNA-seq. (XLSX)

S4 Table Minimal data set. (XLSX)

S1 Fig Gene ontology analysis for the upregulated-gene list. Using clusterprofiler, Fisher’s exact probability test was performed for terms registered in GO (BP). A balloonplot was created by sorting the terms in order of increasing the gene ratio (count data / number of differentially expressed genes).
(TIFF)

S2 Fig Gene ontology analysis for the downregulated-gene list. (TIFF)

S3 Fig Pathway analysis for the upregulated-gene list. Using clusterprofiler, Fisher’s exact probability test was performed for terms registered in wiliPathways. A balloonplot was created by sorting the terms in order of increasing the gene ratio (count data / number of differentially expressed genes).
(TIFF)

S4 Fig Pathway analysis for the downregulated-gene list. (TIFF)

S5 Fig Discrimination between WT and HIF3A_ins transcripts. Total RNA was extracted from the adult lungs of both WT and HIF3A_ins mice. For cDNA synthesis, five hundred ng of total RNA were used with SuperScript™ IV VILO. We designed a specific probe to discriminate between the WT and HIF3A_ins transcripts using the Custom TaqMan™ SNP Genotyping Assay, non-human (Applied BiosystemsTM, Waltham, MA, USA). Quantitative PCR was conducted using 2x TaqMan Master Mix and our custom TaqMan primer probes (Assay ID: ANYMYJ7). Normalized reporter was calculated by dividing the fluorescence signal of the reporter dye (FAM or VIC) by the fluorescence signal of the passive reference dye (ROX).
(TIF)

S6 Fig Discrimination between WT and HIF3A_del transcripts. Total RNA was extracted from the adult lungs of both WT and HIF3A_del mice. We designed a specific probe to discriminate between the WT and HIF3A_del transcripts using the Custom TaqMan™ SNP Genotyping Assay, non-human (Applied Biosystems^TM^, Waltham, MA, USA). Quantitative PCR was conducted using our custom TaqMan primer probes (Assay ID: ANZTT44).
(TIF)

S7 Fig Representative micrographs of Elastica van Gieson staining of 12-week-old mice. The number of blood vessels surrounded by elastin fibers was counted in Elastica van Gieson-stained sections, and the morphology of pulmonary microvessels (<30 μm diameter excluding capillaries) was evaluated as single elastin fiber (SEF), partial elastin fiber (PEF), or multiple elastin fiber (MEF). Frequency of each vessel type was evaluated in at least three independent 12-week-old mice of each genotype. Red arrow shows partially doubled elastin layers. Scale bars indicate 30 μm.
(PDF)

S8 Fig Representative images of the heart in 12-week-old mice. Hematoxylin-eosin staining of sections from WT, HIF3A_ins and HIF3A_del. Scale bars indicate 500 μm.
(PDF)

S1 Appendix Sequence data of WT, HIF3A_ins, and HIF3A_del transcripts. (ZIP)

S2 Appendix Whole gene expression profile analyzed by RNA-seq. (ZIP)

S1 Raw image (PDF)
