# Supplementary figures and images for: HIF3A gene disruption causes abnormal alveoli structure and early neonatal death
Source: PLoS One. 2024 May 8;19(5):e0300751. doi: 10.1371/journal.pone.0300751 (PMC11078382; doi:10.1371/journal.pone.0300751)

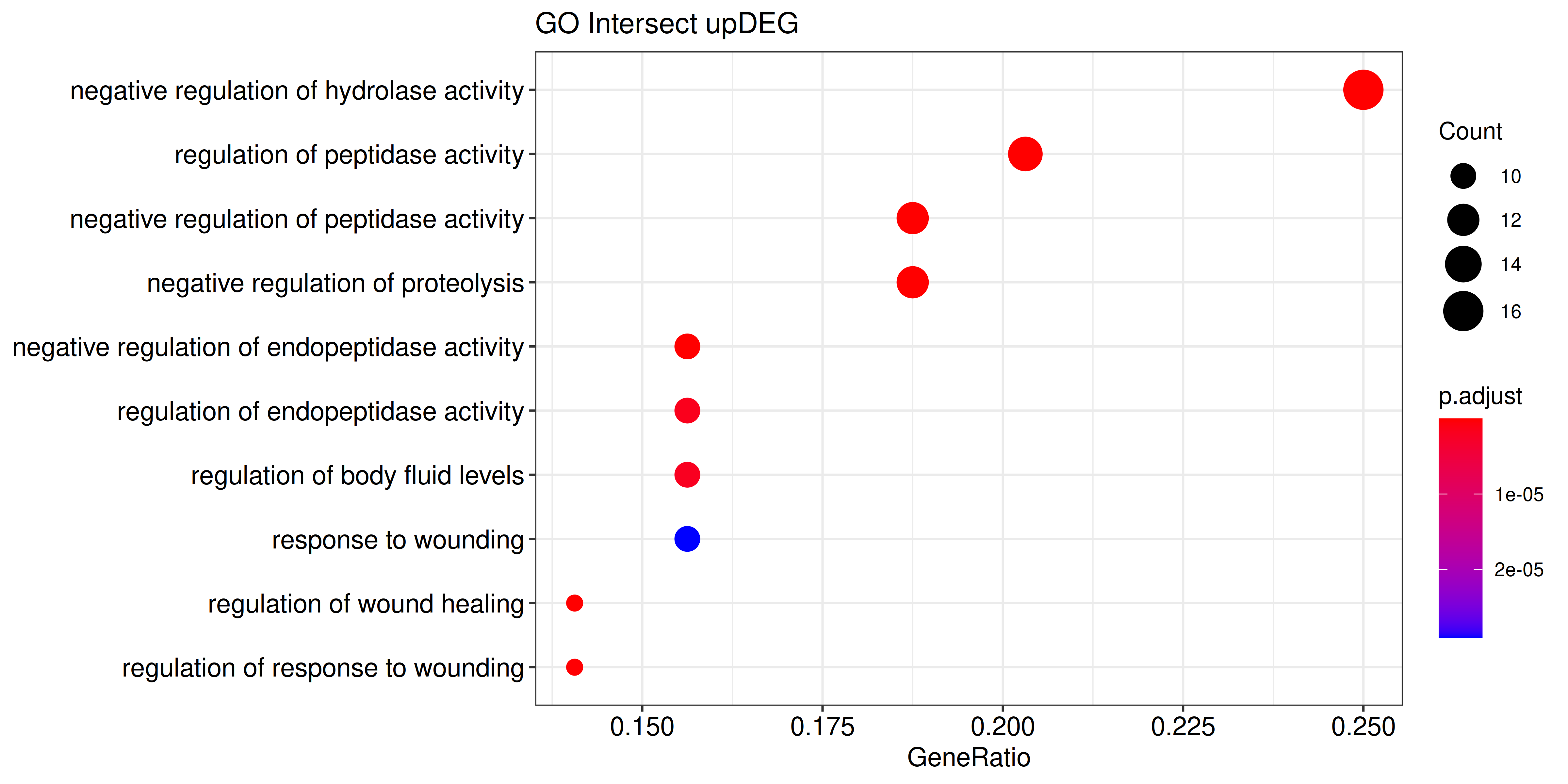

Supplement: S1 Fig — Using clusterprofiler, Fisher’s exact probability test was performed for terms registered in GO (BP). A balloonplot was created by sorting the terms in order of increasing the gene ratio (count data / number of differentially expressed genes). (TIFF) [file pone.0300751.s005.tiff]

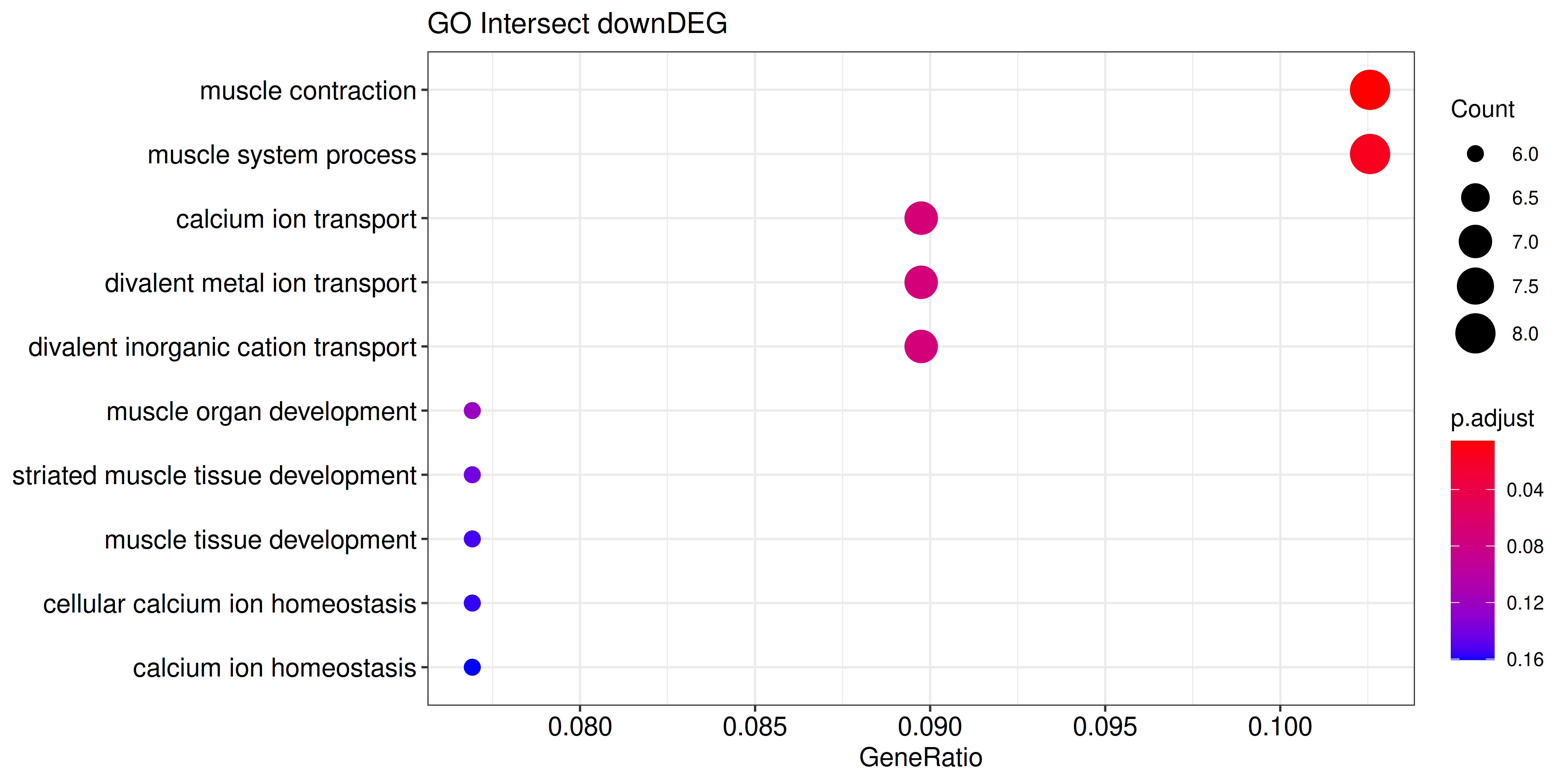

Supplement: S2 Fig — (TIFF) [file pone.0300751.s006.tiff]

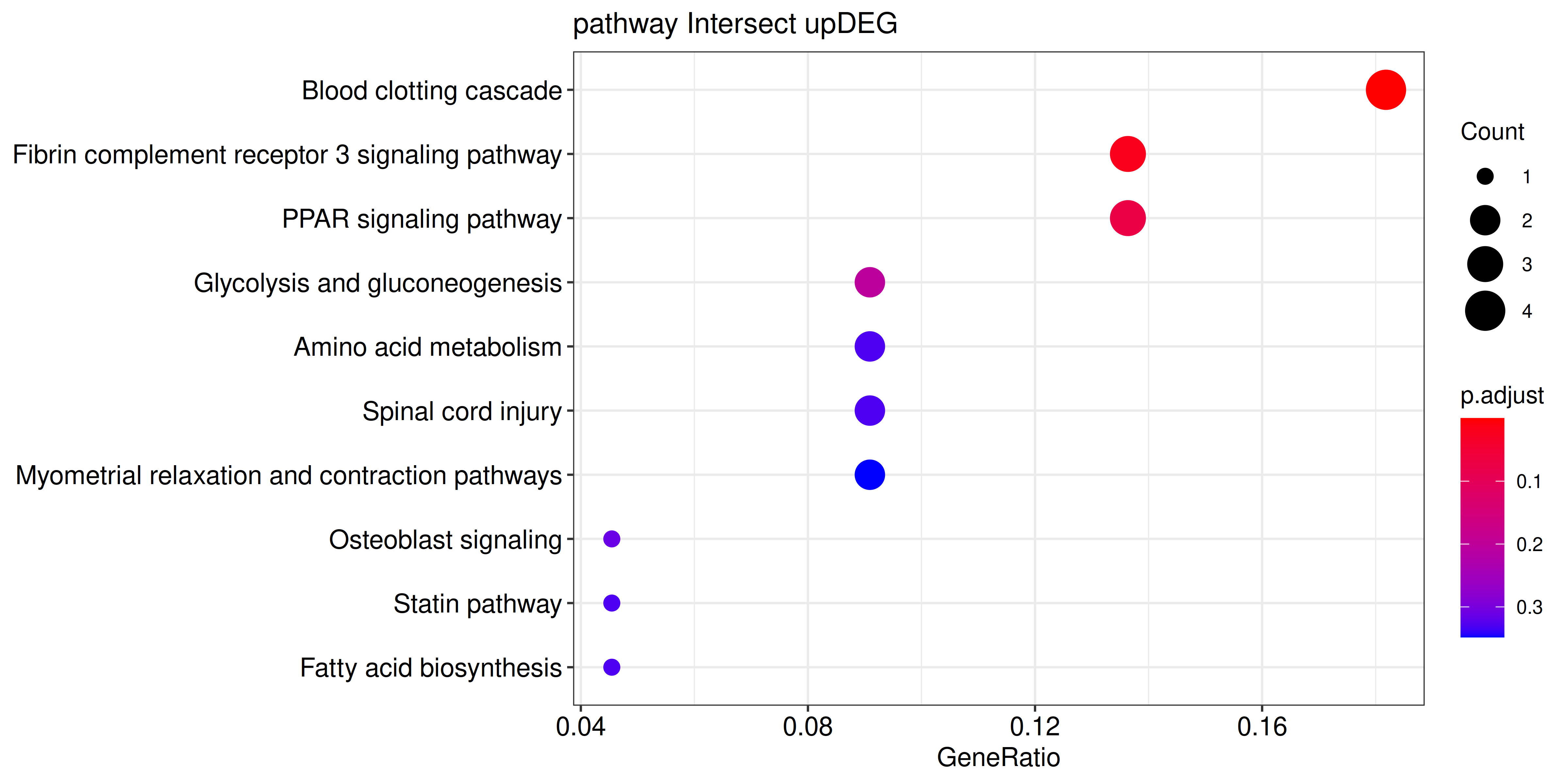

Supplement: S3 Fig — Using clusterprofiler, Fisher’s exact probability test was performed for terms registered in wiliPathways. A balloonplot was created by sorting the terms in order of increasing the gene ratio (count data / number of differentially expressed genes). (TIFF) [file pone.0300751.s007.tiff]

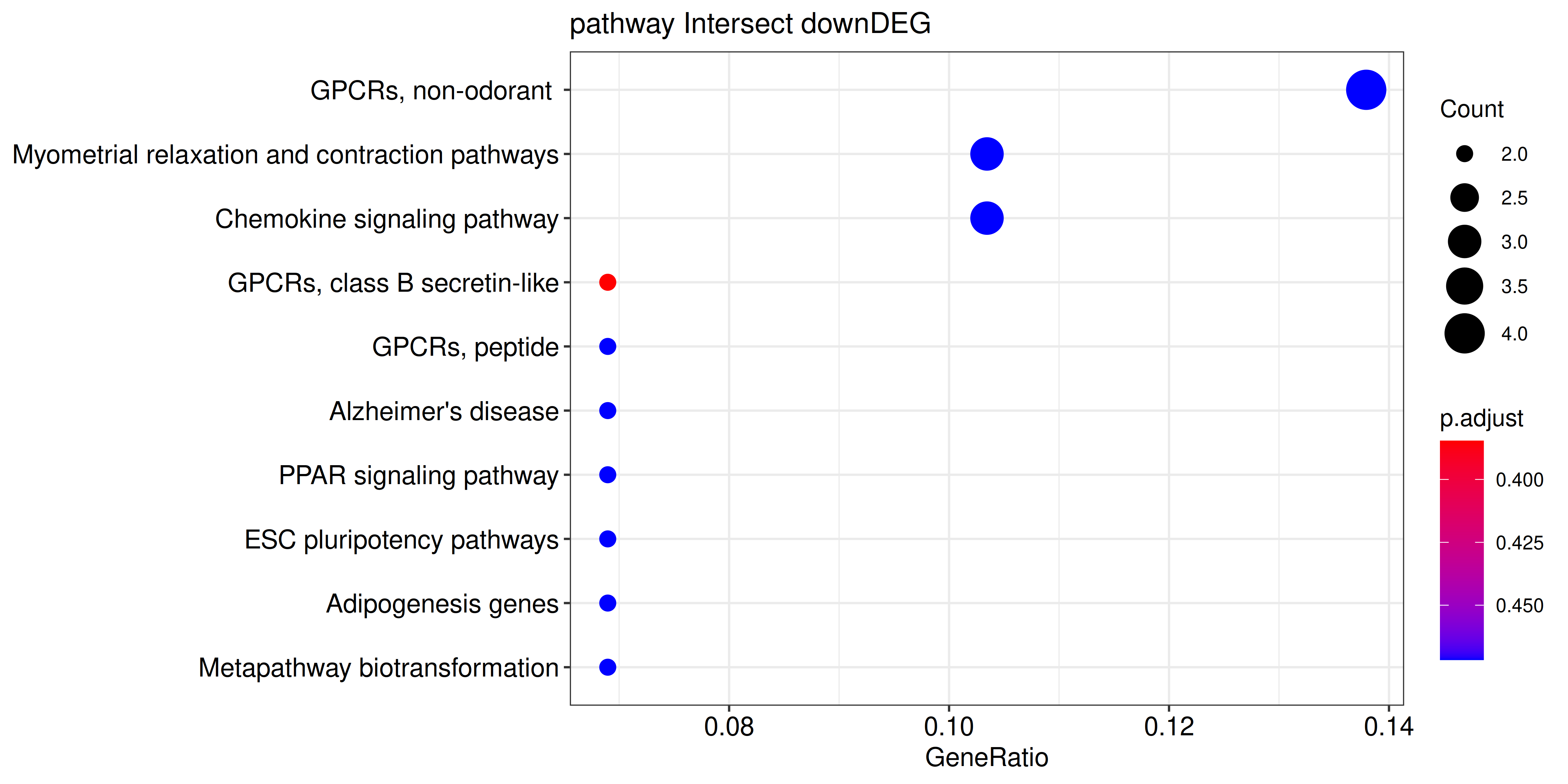

Supplement: S4 Fig — (TIFF) [file pone.0300751.s008.tiff]

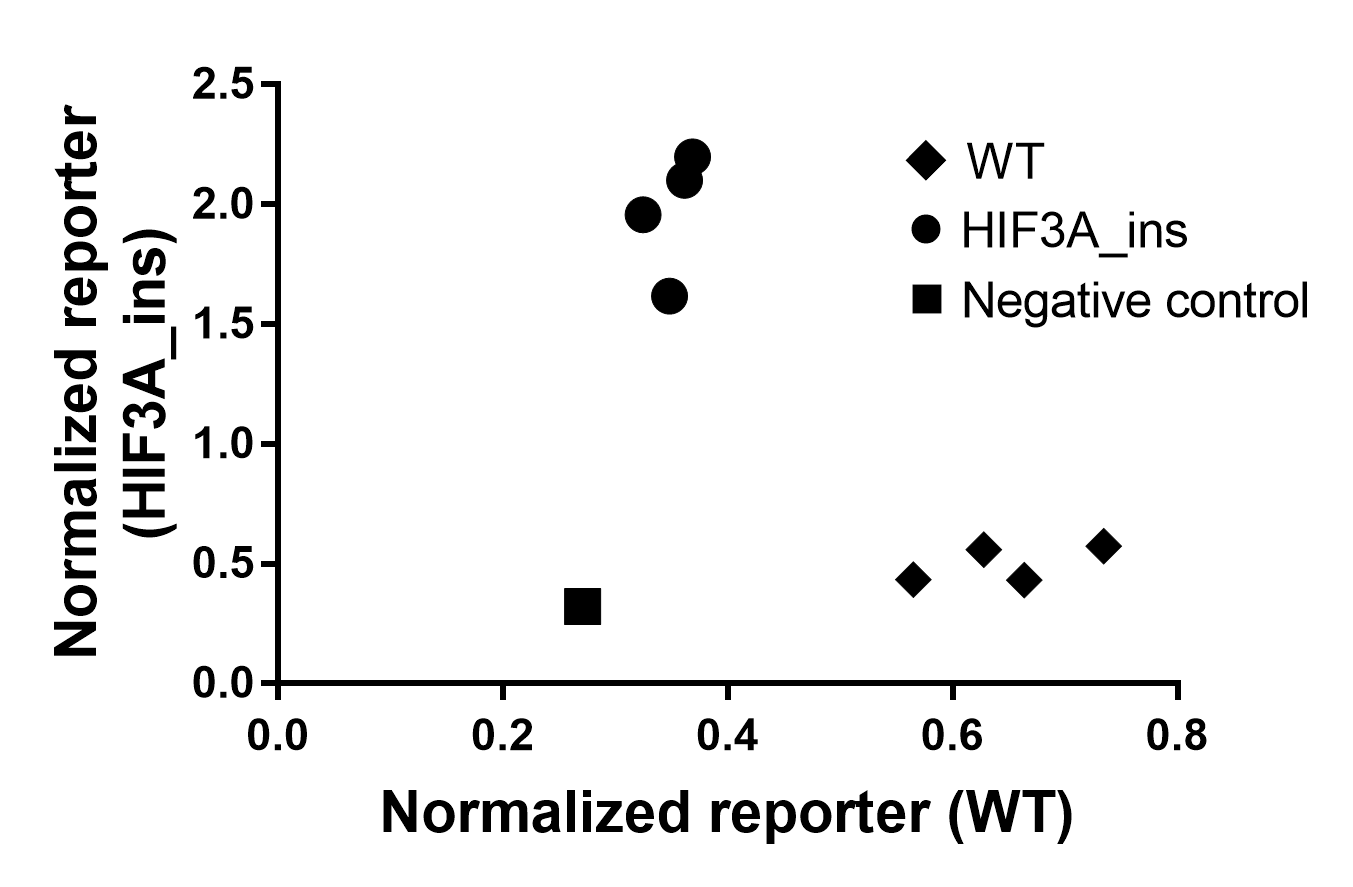

Supplement: S5 Fig — Total RNA was extracted from the adult lungs of both WT and HIF3A_ins mice. For cDNA synthesis, five hundred ng of total RNA were used with SuperScript™ IV VILO. We designed a specific probe to discriminate between the WT and HIF3A_ins transcripts using the Custom TaqMan™ SNP Genotyping Assay, non-human (Applied BiosystemsTM, Waltham, MA, USA). Quantitative PCR was conducted using 2x TaqMan Master Mix and our custom TaqMan primer probes (Assay ID: ANYMYJ7). Normalized reporter was calculated by dividing the fluorescence signal of the reporter dye (FAM or VIC) by the fluorescence signal of the passive reference dye (ROX). (TIF) [file pone.0300751.s009.tif]

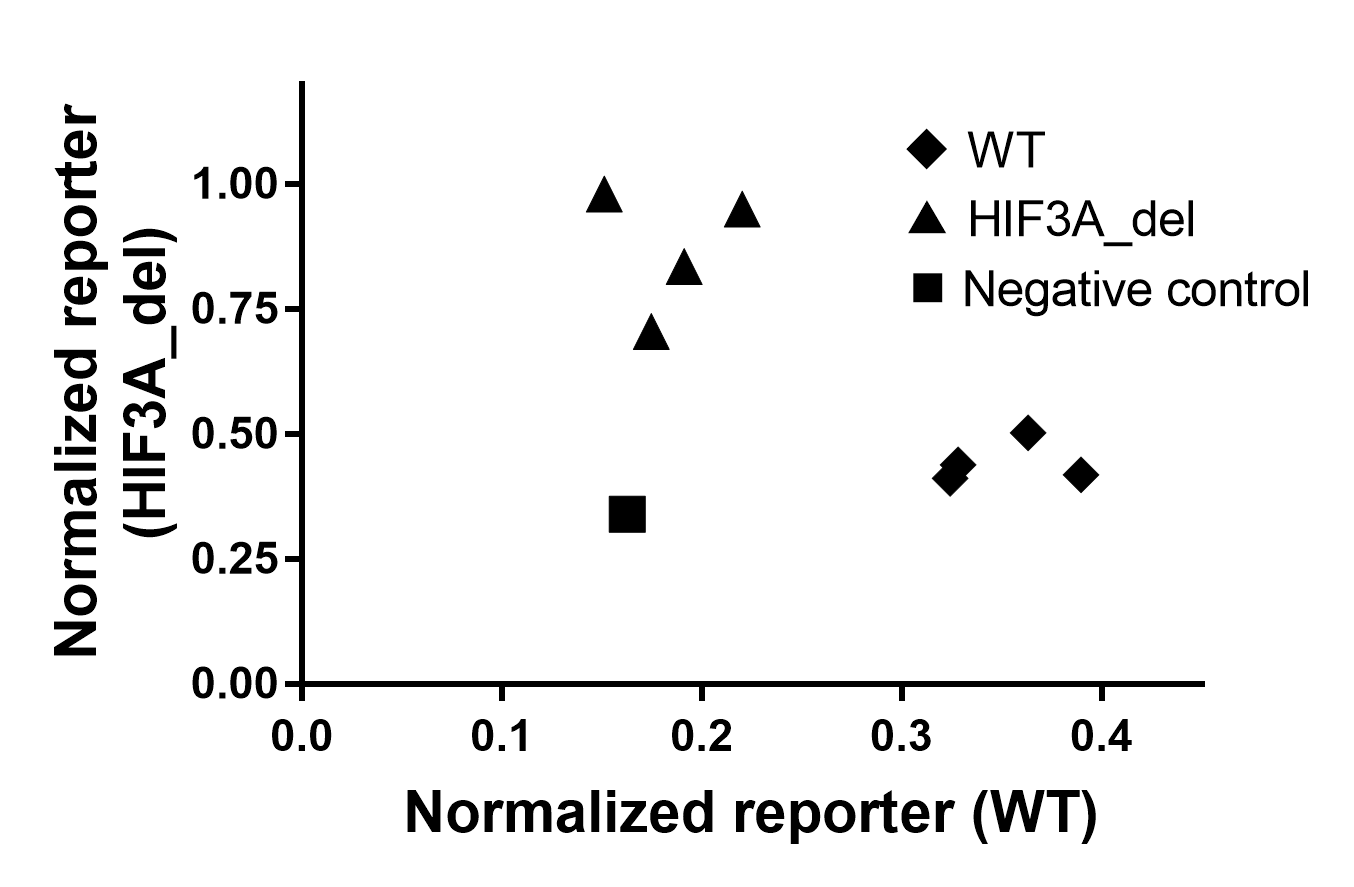

Supplement: S6 Fig — Total RNA was extracted from the adult lungs of both WT and HIF3A_del mice. We designed a specific probe to discriminate between the WT and HIF3A_del transcripts using the Custom TaqMan™ SNP Genotyping Assay, non-human (Applied BiosystemsTM, Waltham, MA, USA). Quantitative PCR was conducted using our custom TaqMan primer probes (Assay ID: ANZTT44). (TIF) [file pone.0300751.s010.tif]

S7 Fig.

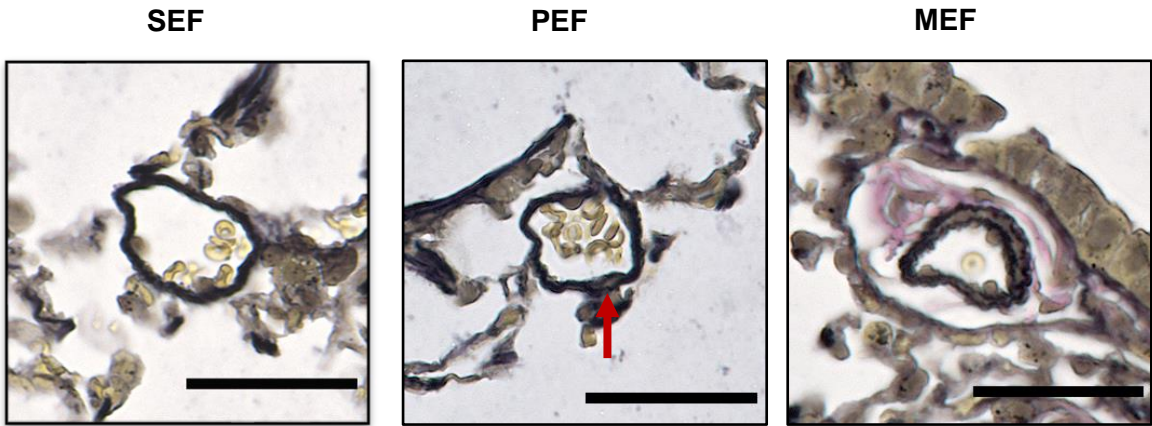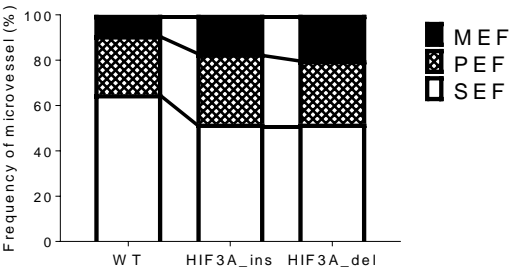

Supplement: S7 Fig — The number of blood vessels surrounded by elastin fibers was counted in Elastica van Gieson-stained sections, and the morphology of pulmonary microvessels (<30 μm diameter excluding capillaries) was evaluated as single elastin fiber (SEF), partial elastin fiber (PEF), or multiple elastin fiber (MEF). Frequency of each vessel type was evaluated in at least three independent 12-week-old mice of each genotype. Red arrow shows partially doubled elastin layers. Scale bars indicate 30 μm. (PDF) [file pone.0300751.s011.pdf]

**S8 Fig.**

**WT**

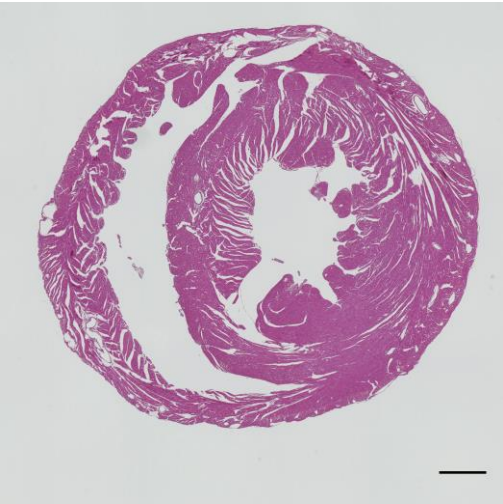

**HIF3A<sub>ins</sub>**

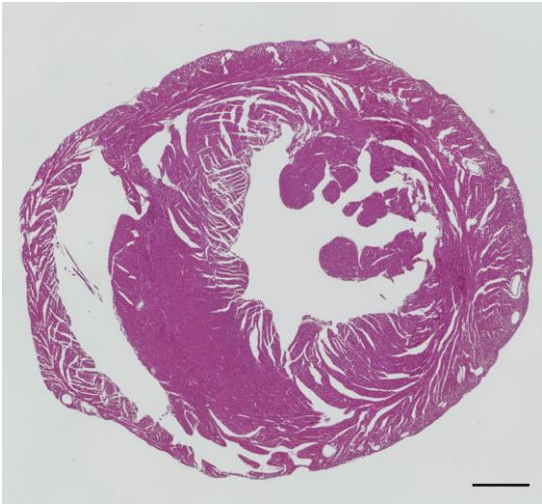

**HIF3A<sub>del</sub>**

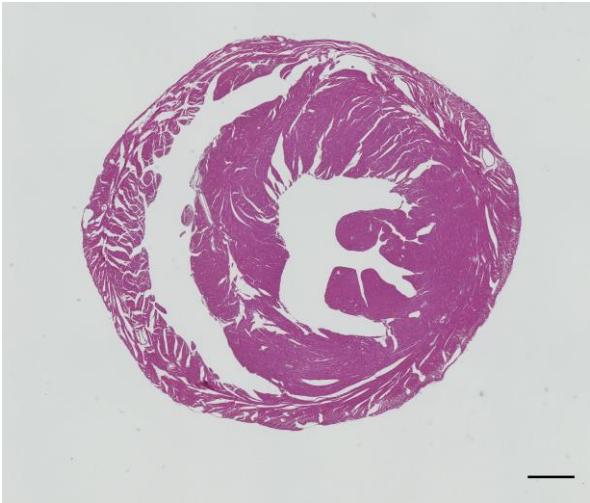

Supplement: S8 Fig — Hematoxylin-eosin staining of sections from WT, HIF3A_ins and HIF3A_del. Scale bars indicate 500 μm. (PDF) [file pone.0300751.s012.pdf]

Figure 1 (C)

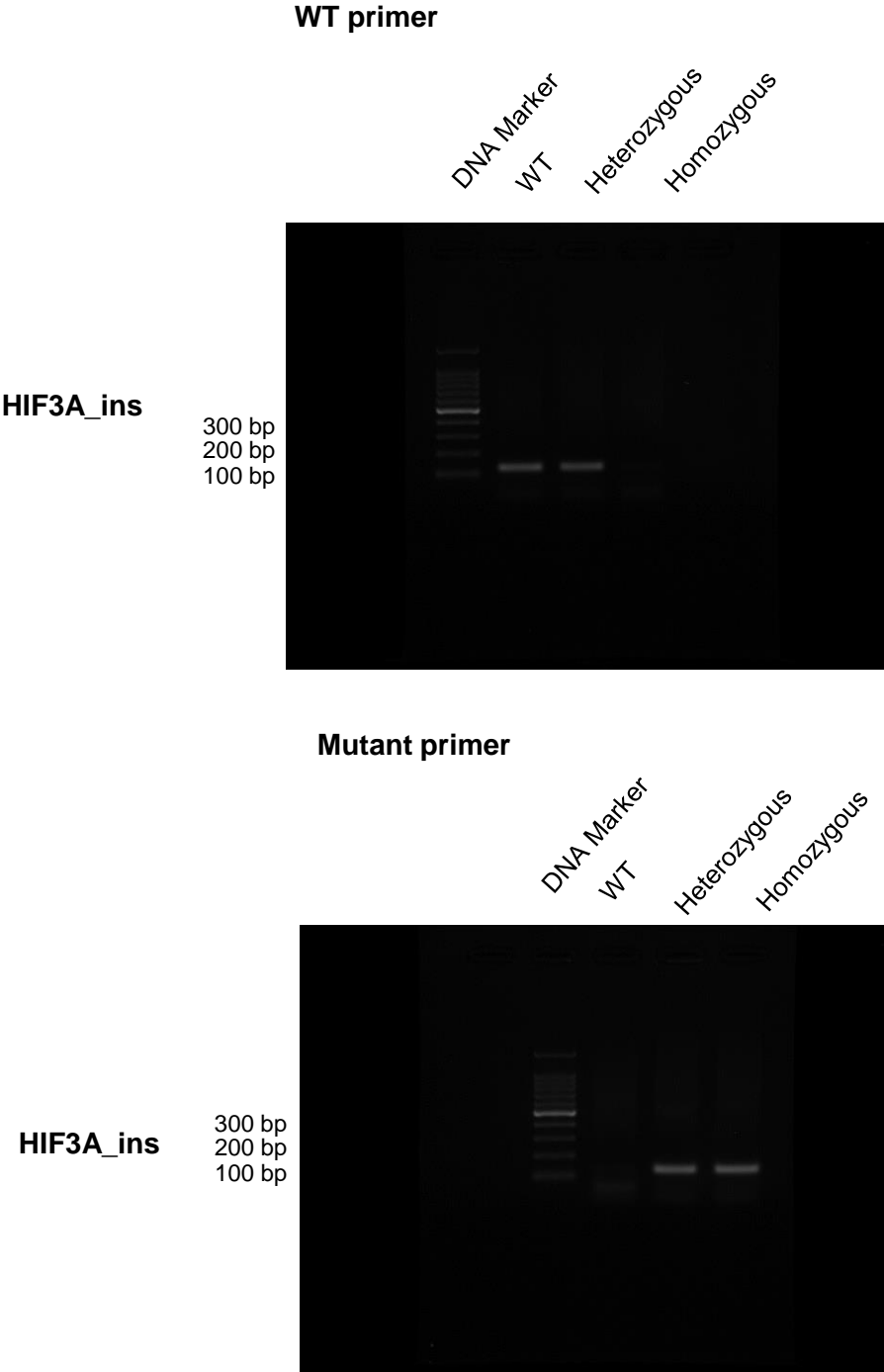

**Figure 1 (C)**

**WT primer**

**HIF3A\_del**

300 bp  
200 bp  
100 bp

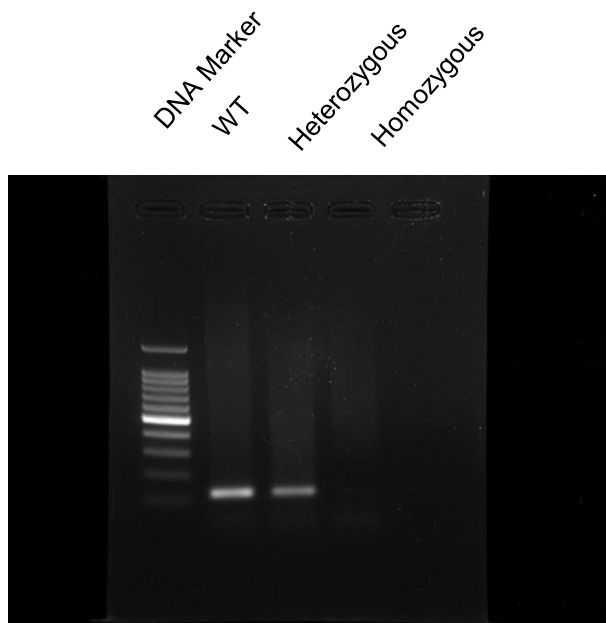

**Mutant primer**

**HIF3A\_del**

300 bp  
200 bp  
100 bp

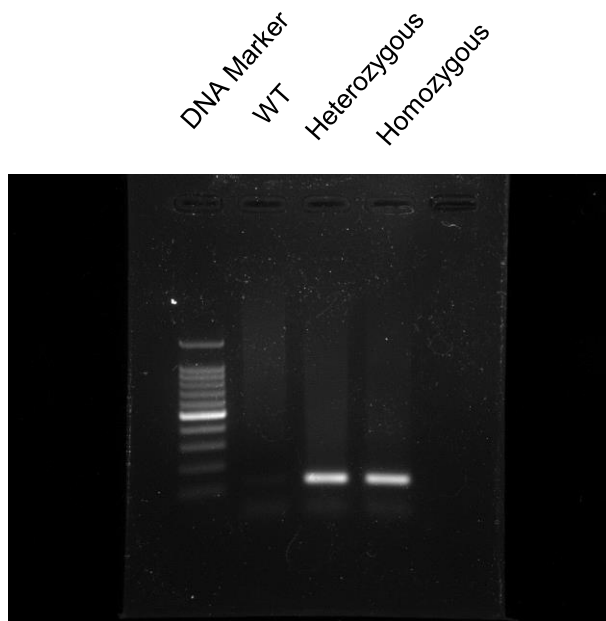

Supplement: S1 Raw image — (PDF) [file pone.0300751.s015.pdf]
